# Enantiomerically pure amino-alcohol quinolines: *in vitro* anti-malarial activity in combination with dihydroartemisinin, cytotoxicity and *in vivo* efficacy in a *Plasmodium berghei* mouse model

**DOI:** 10.1186/1475-2875-13-407

**Published:** 2014-10-16

**Authors:** Catherine Mullié, Nicolas Taudon, Camille Degrouas, Alexia Jonet, Aurélie Pascual, Patrice Agnamey, Pascal Sonnet

**Affiliations:** Equipe Théra - Laboratoire de Glycochimie, des Antimicrobiens et des Agroressources (LG2A) FRE-CNRS 3517, Université de Picardie Jules Verne, UFR de Pharmacie, 1 rue des Louvels, 80037 Amiens Cedex 1, France; UMR-MD3, Institut de Recherche Biomédicale des Armées, BP 73, 91223 Brétigny-sur-Orge, France; UMR-MD3, Institut de Recherche Biomédicale des Armées, Faculté de Pharmacie, Aix-Marseille Université, 27 Bd Jean Moulin CS30064, 13385 Marseille cedex 5, France; Département d’Infectiologie de Terrain, Unité de Parasitologie, Institut de Recherche Biomédicale des Armées, Marseille, France; Laboratoire de Parasitologie et Mycologie, Amiens University Hospital, Avenue Laënnec, 80054 Amiens, France

**Keywords:** Malaria, *Plasmodium falciparum*, *Plasmodium berghei*, *in vivo*, Anti-malarial activity, Quinoline, Enantiomer, Dihydroartemisinin, Isobologram, Combination

## Abstract

**Background:**

As resistance to marketed anti-malarial drugs continues to spread, the need for new molecules active on *Plasmodium falciparum*-resistant strains grows. Pure (*S*) enantiomers of amino-alcohol quinolines previously displayed a good *in vitro* anti-malarial activity. Therefore, a more thorough assessment of their potential clinical use through a rodent model and an *in vitro* evaluation of their combination with artemisinin was undertaken.

**Methods:**

Screening on a panel of *P. falciparum* clones with varying resistance profiles and regional origins was performed for the (*S*)-pentyl and (*S*)-heptyl substituted quinoline derivatives, followed by an *in vitro* assessment of their combination with dihydroartemisinin (DHA) on the 3D7 clone and an *in vivo* assay in a mouse model infected with *Plasmodium berghei*. Their haemolytic activity was also determined.

**Results:**

A steady anti-malarial activity of the compounds tested was found, whatever the resistance profile or the regional origin of the strain. (*S*)-quinoline derivatives were at least three times more potent than mefloquine (MQ), their structurally close parent. The *in vitro* combination with DHA yielded an additive or synergic effect for both that was as good as that of the DHA/MQ combination. *In vivo*, survival rates were similar to those of MQ for the two compounds at a lower dose, despite a lack of clearance of the parasite blood stages. A 50% haemolysis was observed for concentrations at least 1,000-fold higher than the antiplasmodial IC_50_s.

**Conclusions:**

The results obtained make those two (*S*)-amino-alcohol quinoline derivatives good candidates for the development of new artemisinin-based combination therapy (ACT), hopefully with fewer neurologic side effects than those currently marketed ACT, including MQ.

## Background

The latest figures on the incidence and mortality of malaria show that, despite progress in the implementation of preventive measures such as insecticide-treated mosquito nets and intermittent preventive treatments, this parasitic disease is still estimated to affect over 207 million people and to account for 627,000 deaths in 2012. The death toll is particularly high in children under five and pregnant women of the World Health Organization (WHO) African region [1]. Even though the proportion of *Plasmodium vivax* cases rises in certain regions, the vast majority of malaria cases and deaths are due to *Plasmodium falciparum* infections [1]. This species has elaborated resistance mechanisms against almost all anti-malarial drugs available on the market today [2]. Even artemisinin derivatives have seen their efficacy challenged in Southeast Asia and, more recently, in South America [3, 4], leading WHO to officially recommend the use of artemisinin-based combination therapy (ACT) as first-line treatment of uncomplicated malaria as far back as in 2006 [5]. This recommendation was reiterated in the 2011 WHO Global Plan for artemisinin resistance containment [6].

**Figure 1:**
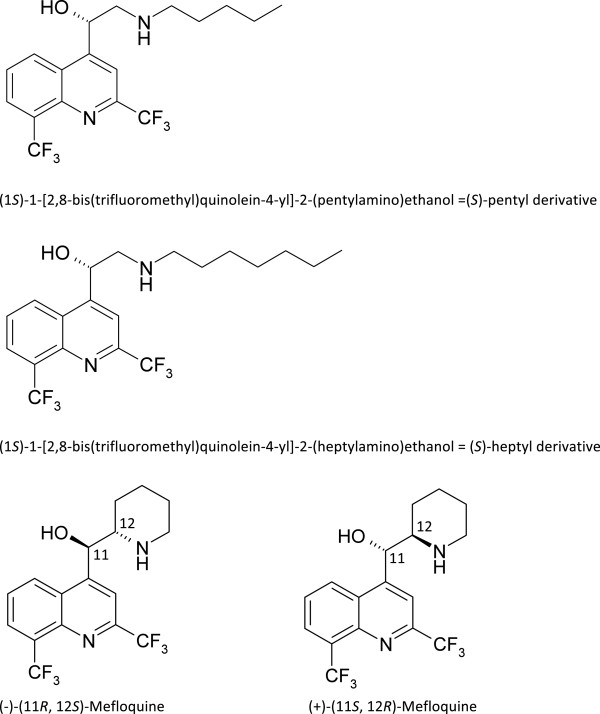
**Structure of the 4-amino-alcohol quinolines used in this study.**

One such ACT currently in use is the combination of artemisinin derivatives with mefloquine (MQ). This latter molecule is marketed as a racemate of its *erythro* form (Figure 1) and possesses a long half-life (*circa* 14 days) that can be seen as a therapeutic advantage as a lower rate of relapses has been reported for anti-malarials with long half-lives [7, 8]. However, dose-related neuropsychiatric adverse effects have been reported under MQ use, therefore contra-indicating it in individuals with a history of epilepsy or psychiatric disease [9, 10]. As the (+)-enantiomer of MQ was shown to be at least as active as the (−)-enantiomer [11, 12] and less likely to cause toxicity in the central nervous system through the blockage of central adenosine receptors [13], a possible way to circumvent some of the neurotoxicity of MQ has been envisaged through the synthesis of a series of enantiomerically pure MQ amino analogues [14]. Their anti-malarial activity against *P. falciparum* W2 and 3D7 strains has also been documented, showing that (*S*) enantiomers were more active than their (*R*) counterparts by a factor ranging from 2 to 15, depending on the side chain length [15]. The aim of this work was therefore to build on these first observations by choosing the two most effective molecules in the series, the (*S*)-pentyl and (*S*)-heptyl substituted amino-alcohol quinolines (Figure 1). First, the assessment of their activity on a wider range of *P. falciparum* clones, coming from various origins and exhibiting different resistance profiles to anti-malarial drugs in use, was carried out to ensure they could be of use against the vast majority of *P. falciparum* clones. Then, the two molecules were assessed in a mouse model of *Plasmodium berghei* infection to further validate their potential as drug candidates, in parallel with a screening of their potential cytotoxicity. Finally, their anti-malarial efficacy in combination with one of artemisinin derivatives, dihydroartemisinin (DHA), was checked to ascertain whether such a combination would be of relevant clinical use.

## Methods

### Chemical products

The test drugs included DHA, chloroquine (CQ) diphosphate, MQ hydrochloride (Sigma-Aldrich, Saint-Quentin Fallavier, France) and the 4-amino-alcohol quinolines represented in Figure 1. The 4-amino-alcohol quinolines were synthesized following the general procedure described by Jonet *et al.* [14]. Only their (*S*) enantiomers were used as they had previously been shown to be more potent than their (*R*) analogues [15]. All other chemical products were purchased from Sigma-Aldrich (Saint-Quentin-Fallavier, France), unless stated otherwise.

### *Plasmodium falciparum*culture

Parasites were cultivated in A^+^ human erythrocytes (2% haematocrit) suspended in RPMI 1640 medium (Invitrogen, Paisley, UK) supplemented with 10% human serum (Abcys S A, Paris, France) and buffered with 25 mM HEPES-25 mM NaHCO_3_ under controlled atmospheric conditions (10% O_2_, 5% CO_2_, and 85% N_2_) at 37°C with 95% humidity [16].

### Drug sensitivity assay on selected *Plasmodium falciparum*clones

The anti-malarial activity of the (*S*)*-*pentyl and (*S*)*-*heptyl substituted amino-alcohol quinoline derivatives was tested against a panel of strains or clones depicted in Table 1. All strains were twice synchronized with D-sorbitol 5% (Fluka, Saint Quentin Fallavier, France) before the assay [17]. CQ and MQ were routinely included as positive controls as well as negative controls using solvent (water, dimethyl sulphoxide or methanol, depending on the drug) .

**Table 1:** **Susceptibility and geographical origin of the** ***Plasmodium falciparum*** **strains and clones used in this study**

Strain/Clone	Geographical origin	Chloroquine susceptibility	Mefloquine susceptibility
K1	Thailand	R^a^	S
W2	Indochina	R	S
FCM29	Cameroon	R	S
3D7	From NF54 African strain (MR4: Malaria Research and Reference Reagent Resource centre)	S	R
HB3	Honduras	S	R
BRE1	Brazil	R	R
Dd2	Indochina (from W2 strain)	R	R

^a^:Parasites were considered resistant (R) if their IC_50_ was higher than 100 nM for chloroquine and 50 nM for mefloquine.

The 50% inhibitory concentration (IC_50_) was evaluated using tritiated hypoxanthine [16]. The specific activity of tritiated hypoxanthine is 1 mCi/mL (Perkin-Elmer, Courtaboeuf, France). The IC_50_ values were evaluated by analysing incorporation of tritiated hypoxanthine according to the concentration by a non-linear regression analysis processing on dose–response curves (RiaSmart, Packard, Meriden, USA). Results were expressed as geometrical average of IC_50_.

### *In vitro*combination assay

*Plasmodium falciparum* parasite strain 3D7 was used for this experiment. MQ, the (*S*)-pentyl and (*S*)-heptyl derivatives were associated with DHA following the fixed-ratio method of Fivelman *et al.* [18]. All compounds were solubilized in dimethyl sulphoxide (DMSO) to prepare extemporaneous stock solutions. DHA and either the (*S*)-pentyl, (*S*)-heptyl derivative or MQ were then mixed to reach final ratios of 5:0, 4:1, 3:2, 2:3, 1:4, and 0:5. The mixed stock solutions were then diluted in supplemented RPMI medium so as to reach eight times the IC_50_ previously determined for the 3D7 strain in the most concentrated well for the 5:0 mix of each compound. Serial two-fold dilutions were then carried out in 96-well-plates under a 50 μL final volume to generate a range of six concentrations for each mix. The final concentration of DMSO in the wells never exceeded 0.02% (v/v). A negative control with the same amount of DMSO was performed to check for any potential toxicity. Then, 200 μL of parasitized red blood cells (pRBC) (final parasitaemia of 0.5% and final haematocrit of 2%) were added. After a 48-hour incubation at 37°C with the drugs, growth inhibition was assessed and dose–response curves fitted.

The assessment of drug interaction was based on the calculation of the fractional inhibitory concentrations (FICs) of the two molecules. The FIC was calculated for each association by dividing the IC_50_ of the drug in the combination by the IC_50_ of the drug alone. The sum of these two FICs (∑FICs) was calculated to plot isobologram curves [18, 19] using the software R [20]. ΣFICs <1 denote synergism, ΣFICs ≥1 and <2 denote additive interaction, ΣFICs ≥2 and <4 denote slight antagonism, and ΣFICs ≥4 denote marked antagonism. ΣFICs <0.5 indicate substantial synergism [19].

### *In vivo*assay

The BALB/c female mice used in this assay were four weeks old and pathogen-free (Charles River Laboratories, France). They were housed under standard conditions, with unlimited access to food and water. All experiments adhered to the French guidelines for animal research and were approved by the ethical committee of the Institut de Recherche Biomédicale des Armées - Antenne de Marseille (Number 2007–02). All efforts were made to minimize animal suffering.

The *P. berghei* ANKA line was graciously provided by Dr Salah Méchéri (Institut Pasteur, Paris, France). Mice (average body weight 16.7 g) were infected with *P. berghei* parasites by intraperitoneal (IP) inoculation of donor mouse blood diluted in normal saline so as to contain 3.10^6^ infected red blood cells (RBC). Parasitaemia was then monitored regularly with blood smears. The treatment was started when the parasitaemia of receiver mice was greater than 1% (Day 0). Five groups of seven mice were randomly distributed. These groups received a daily IP dose for five days of either MQ at 9 mg/kg or either the (*S*)-pentyl or (*S*)-heptyl derivative (hydrochloride salts) at 3 or 9 mg/kg under a volume of 100 μL per 20 g of body weight. A control group (13 mice) received an IP of the vehicle. Afterwards, the parasitic growth was evaluated daily by blood smears and mice were monitored for their survival up to Day 40.

The parasite multiplication rate (PMR) was used to normalize the activity of the studied compounds on the parasite growth (treated), to the natural parasite growth (control) [21].

Survival curves were used to study the likelihood of death in treated and control mice. They were fitted using GraphPad Prism® software version 5.04 (GraphPad Software Inc, 2012, La Jolla, CA). The survival analysis was performed according the method of Kaplan-Meier, which determines the probability of survival when at least one ‘death’ is recorded [22].

### Haemolysis test

The assay was adapted from the protocol described by Taniyama *et al*. [23]. Briefly, human RBC were centrifuged (1,000 g, 10 min), the pellet washed three times with TRIS buffer (TRIS 10 mmol.L^−1^, NaCl 150 mmol.L^−1^, pH 7.4) and finally diluted at 0.5% (v/v) in the same buffer. Experiments were conducted in triplicate on 96-well plates. Saponin 1% (m/v) was used as a positive haemolysis control (100% haemolysis), DMSO and TRIS buffer were used as negative controls. Fifteen μL of the tested molecule (concentration range: 120 nmol.L^−1^ to 500 μmol.L^−1^) or control solution were added to 235 μL of the 0.5% (v/v) RBC suspension. Plates were incubated at 37°C for 1 hour and then centrifuged (1,000 g, 10 min). Then, 100 μL of supernatant were retrieved from each well for optical density measurement at 405 nm. The percentage of haemolytic activity was calculated using the saponin mean absorbance at 405 nm as the 100% haemolysis value and the negative control absorbance at 405 nm as blank. The drug concentration inducing a 50% haemolysis (HC_50_) was then deduced from the dose–response curves.

### Statistical analysis

The Pearson’s correlation coefficient between IC_50_s values obtained on the various strains for CQ, MQ and the (*S*)-pentyl derivative was calculated and the test for the statistical significance of the obtained value of r subsequently performed. A log-rank test was performed to compare the survival and the delay for parasitic recrudescence curves. A two-way analysis of variance (ANOVA) on treatment and time parameters was performed to compare PMR results, using the R software [20]. A p-value inferior to 0.05 was considered as significant.

## Results

### *In vitro*anti-malarial activity against selected *Plasmodium falciparum*strains/clones

The results obtained for CQ, MQ, the (*S*)-pentyl, and (*S*)-heptyl derivatives are summarized in Table 2. A one-way ANOVA was first carried out and indicated a significant difference in IC_50_s between *P. falciparum* clones (p = 1.7 10^−8^) for MQ. Post-ANOVA tests confirmed the differences in IC_50_s between clones K1 and FCM29 (p = 0.02), FCM29 and 3D7 (p = 4.48 10^−4^), 3D7 and Dd2 (p = 0.0173). A two-way ANOVA, taking into account both the strain and the molecule tested, pointed towards a significant difference between groups (p = 5.45 10^−14^).

**Table 2:** **IC**_**50**_ **s expressed as average (CV%) for CQ, MQ, (** ***S*** **)-pentyl and (** ***S*** **)-heptyl derivatives on the selected** ***Plasmodium falciparum*** **strains and clones**

	IC _50_ (nmol L ^−1^)				Ratio	
Strain/Clone	CQ	MQ	***S***-pentyl	***S***-heptyl	MQ/( ***S***)-pentyl	CQ/( ***S***)-pentyl
K1	164 (35.1)	14.3 (28.0)	4.1 (21.0)	ND^a^	3.5	40.0
W2	572 (19.5)	26.5 (9.2)	7.0 (8.9)	9.4 (9.7)	3.8	81.7
FCM29	738 (7.2)	24.5 (10.1)	3.7 (10.5)	ND	6.6	199.6
3D7	21.2 (13.7)	67.0 (5.89)	12.8 (3.1)	14.5 (8.5)	5.3	1.7
BRE1	265 (33.0)	72.3 (10.4)	11.7 (6.9)	ND	6.2	22.6
Dd2	169 (20.4)	84.7 (7.8)	5.5 (19.3)	ND	15.9	30.7
HB3	13.8 (20.5)	98.7 (9.8)	13.9 (7.4)	ND	7.1	1.0

^a^:not determined.

These strains were selected to evaluate the efficacy of the (*S*)-pentyl substituted amino-alcohol quinoline against a variety of resistance profiles both to CQ and MQ and, to a lesser extent, the efficiency of the (*S*)-heptyl. The clones were also selected to allow for a variety of geographical origins. As similar results were obtained for both derivatives on the first two strains against which the molecules were tested (3D7 and W2), only the (*S*)-pentyl derivate was thereafter tested on the other strains. The IC_50_s obtained with this compound ranged from 3.7 nmol.L^−1^ (FCM29 clone) to 13.9 nmol.L^−1^ (HB3 clone) and were constantly lower than those observed for CQ and, more interestingly, than those for MQ, their structurally close parent (Table 2). These low IC_50_s were retained whatever the resistance profile exhibited by the strains/clones or their geographical origin. The correlations between the CQ and (*S*)-pentyl IC_50_s and the MQ and (*S*)-pentyl IC_50_s were not statistically significant (p = 0.144 and p = 0.0659, respectively). However, CQ and (*S*)-pentyl IC_50_s tended to display a negative correlation while MQ and (*S*)-pentyl IC_50_s tended to be positively correlated (r = −0.612 and r = 0.724, respectively). Nevertheless, the calculated MQ/(*S*)-pentyl IC_50_s ratios varied from 3.5 to 15.9, depending on the strains, giving a clue to an at least partially different mechanism of action for this compound as compared to MQ.

### *In vitro*combination assay

**Figure 2:**
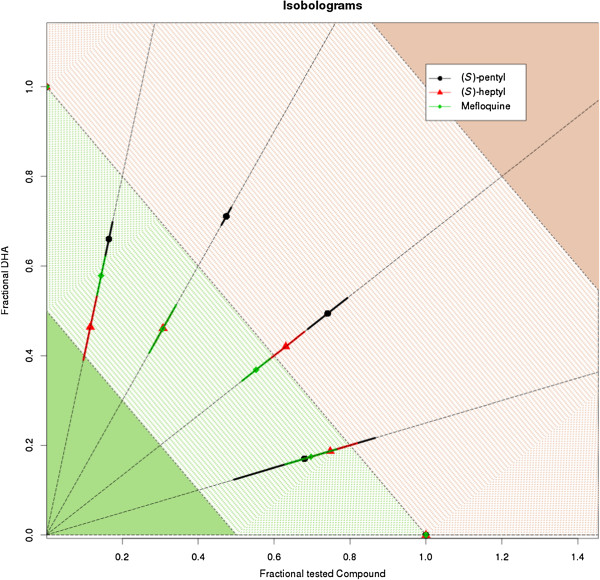
**Isobolograms obtained on the** ***Plasmodium falciparum*** **3D7 clone for the following combinations: DHA/MQ, DHA/** ***S*** **-pentyl derivative, DHA/** ***S*** **-heptyl derivative.** Coloured zones represent synergy, additivity, indifference and antagonism for solid green, dashed green, dashed red and solid red, respectively.

Figure 2 displays the isobolograms resulting from the plotting of ΣFICs obtained on *P. falciparum* 3D7 for the fixed ratios of MQ/DHA, (*S*)-pentyl/DHA and (*S*)-heptyl/DHA. The results observed for the MQ/DHA combination are in the range of those previously reported for example by Gupta *et al*. [24] with a ΣFICs of 0.93 or by Kerschbaumer *et al*. [25] with a ΣFICs of 0.5991 at the IC_50_.

Similar values were obtained for the substituted amino-alcohol quinolines as only the (*S*)-pentyl/DHA association for which the 3:2 and 2:3 ratios exhibited a ΣFIC superior to 1, as well as the 3:2 ratio for the (*S*)-heptyl/DHA combination. Otherwise, ΣFICs for the combinations tested fell between 0.5 and 1, similarly to the ΣFICs obtained for the MQ/DHA combination, denoting a synergistic effect at least as good as that of MQ for the new compounds tested.

### In vivo anti-malarial activity

#### Parasite multiplication rate (PMR)

**Figure 3:**
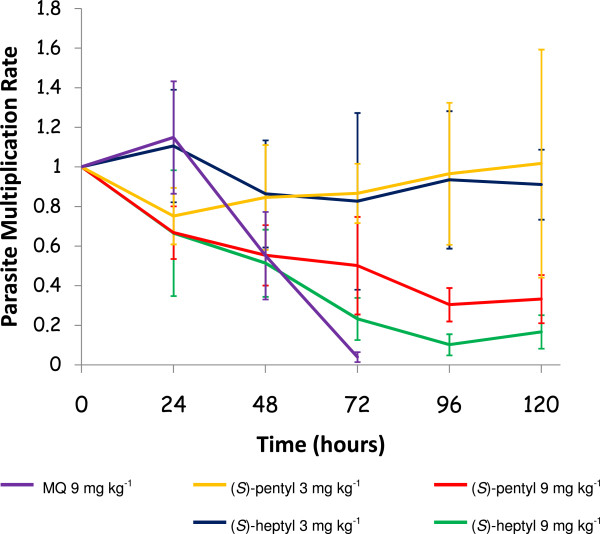
**Parasite multiplication rates of** ***Plasmodium berghei*** **ANKA expressed as mean ± standard error.**

After inoculation, all mice showed positive parasitaemia. When drug administrations were started, an average parasitaemia of 5.2% was observed (95% confidence interval, 1.5-6.0%). As can be seen on Figure 3, at the dose of 9 mg kg^−1^, MQ enabled a clearance of blood parasites within 72 hours. This clearance time is consistent with clinical results obtained in children after a single oral dose of MQ at 25 mg kg^−1^ [26] and in adults after a single 1,000 mg oral dose of MQ [27], although it has to be specified that the administration route was different in these clinical studies as compared to the implemented animal model. The (*S*)-pentyl and (*S*)-heptyl derivatives at 3 mg kg^−1^ did not prevent the parasite multiplication within infected animals. However, at 9 mg kg^−1^, a decrease in the PMR was observed for both compounds, although the clearance of parasites could not be achieved. The statistical analysis (Table 3) indicated the PMR was significantly lower in the MQ group as compared to all of the others. Additionally, a dose effect was observed as the 9 mg kg^−1^ of both amino-alcohol quinoline derivatives was significantly more efficient in reducing the PMR than their 3 mg kg^−1^ dose (Table 3). No significant difference was found between the (*S*)-pentyl and (*S*)-heptyl efficiencies at either 3 or 9 mg kg^−1^.

**Table 3:** **P-values obtained for post-ANOVA two-by-two comparisons on parasite multiplication rates**

		( ***S***)-pentyl		( ***S***)-heptyl	
		3 mg kg ^−1^	9 mg kg ^−1^	3 mg kg ^−1^	9 mg kg ^−1^
(*S*)-pentyl	9 mg kg^−1^	**0.00132** ^**a**^			
(*S)*-heptyl	3 mg kg^−1^	0.0749	0.295		
	9 mg kg^−1^	**5.64 10** ^**−5**^	0.0810	**0.0348**	
MQ	9 mg kg^−1^	**9.47 10** ^**−6**^	**1.62 10** ^**−7**^	**5.02 10** ^**−5**^	**3.49 10** ^**−7**^

^a^:statistically significant values are indicated in bold characters.

#### Survival curves

**Figure 4:**
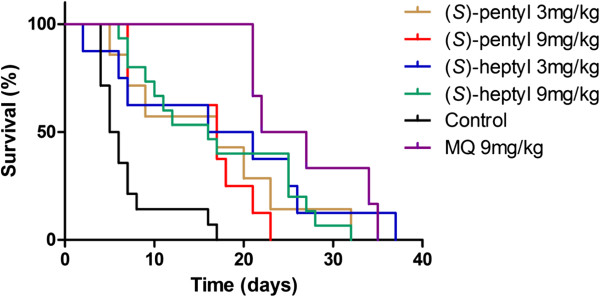
**Survival curves of the BALB/c female mice infected with** ***Plasmodium berghei*** **ANKA.**

The fitted survival curves are presented in Figure 4. The overall test of Mantel Cox emphasized a statistically significant difference between the groups (p <0.0001). The results of the subsequent two-by-two analysis are reported in Table 4. By Day 17, all mice in the control group had died while in treated groups, mice survived significantly longer, whatever the treatment (Table 4). When the survival in the MQ arm is compared to the ones in arms treated with the amino-alcohol quinoline derivatives, a significant difference was only found for the (*S*)-pentyl at 9 mg kg^−1^ arm for which the survival was lower. For the (*S*)-heptyl derivative at the same dose, the difference was not significant. One would have expected the survival curves to be better at the higher concentration tested for amino-alcohol quinoline derivatives. However, some toxicity might explain the difference in survival witnessed *in vivo* for the 9 mg kg^−1^ dose. Interestingly, for either derivative at 3 mg kg^−1^, the survival was as good as the one witnessed for MQ, even though the PMR failed to be reduced at the same concentration. The *in vitro* IC_50_ (*S*)-pentyl/IC_50_ MQ efficiency ratio of 3 and above witnessed on the various *P. falciparum* clones (Table 2) is, therefore, corroborated *in vivo* as similar survival rates were obtained with a dose of derivatives that was one third of the MQ one. However, the PMR did not decline in similar ways with the tested compounds and MQ. The failure to clear the parasites from blood might be due to a slower effect of these molecules, as compared to the one of MQ. Also, the activity of these compounds on sequestered forms, which cannot be investigated by the PMR evaluation, might explain a gain of survival time not associated with a PMR decrease for mice receiving the dose of 3 mg kg^−1^. Along with possible differences in pharmacokinetics, these hypotheses could explain the similar survival rates observed with MQ derivatives, even though the clearance of parasites as measured by the PMR technique was not achieved on Day 5 with these compounds.

**Table 4:** **P-values for differences in survival curves according to the treatment received**

		( ***S***)-pentyl		( ***S***)-heptyl		Mefloquine
		3 mg kg ^−1^	9 mg kg ^−1^	3 mg kg ^−1^	9 mg kg ^−1^	9 mg kg ^−1^
Control		**0.0059** ^a^	**0.0017**	**0.0094**	**0.0002**	**<0.0001**
(*S*)-pentyl	3 mg kg^−1^		0.5691	0.6281	0.8383	0.564
	9 mg kg^−1^			0.2959	0.2382	**0.0071**
(*S*)-heptyl	3 mg kg^−1^				0.8011	0.4406
	9 mg kg^−1^					0.0599

^a^:statistically significant values are indicated in bold characters.

#### Haemolysis test

As a preliminary way to assess the potential cytotoxicity of the (*S*)-pentyl and (*S*)-heptyl derivatives, as they appeared to act on the erythrocytic stage of the parasite cycle, their haemolytic activity was evaluated. The HC_50_ determined for the (*S*)-pentyl and (*S*)-heptyl derivatives were of 302 μmol L^−1^ (95% confidence interval [301.0-302.8]) and 261 μmol L^−1^ (95% confidence interval [235.1-287.9]), respectively. Meanwhile, under the same experimental conditions, MQ displayed an HC_50_ of 203 μmol L^−1^ (95% confidence interval [197.0-210.0]). Therefore, the least favourable HC_50_/IC_50_ ratios calculated for MQ, the (*S*)-pentyl and (*S*)-heptyl derivatives would be of 2,057, 21,726 and 18,000, respectively. As regards the haemolytic activity, the (*S*)-amino-alcohol quinoline derivatives would then have a ten-fold higher safety margin than their MQ parent. An additional point can be made on the safety of use for these products as, in a previous report, the cytotoxicity of MQ and its (*R*)-amino-alcohol derivatives was evaluated on hepatocellular carcinoma HepG2 cells using the MTT assay [28]. Inhibitory concentrations 50, in this case defined as concentrations of products leaving 50% of viable cells as compared to the control, were calculated after a 24-hour incubation with MQ or the amino-alcohol derivatives. The (*R*)-heptyl derivative IC_50_ value was found to be of 16.6 μg mL^−1^ (39.3 μmol L^−1^). MQ gave an IC_50_ value of 11 μg mL^−1^ (26.5 μmol L^−1^). The (*S*)-heptyl derivative, but not the (*S*)-pentyl one, was tested at the same time and yielded an IC_50_ of 26.2 μg mL^−1^/66.5 μmol L^−1^ (95% confidence interval [19.9-32.6 μg mL^−1^]/[50.5-82.7 μmol L^−1^]). The safety indexes of MQ and the (*S*)-heptyl derivative calculated on this model were of 268 and 5,752, respectively. Based on these results, the enantiomerically pure amino-alcohol quinoline derivatives would be at least as safe to use as MQ.

## Conclusion

The (*S*)-pentyl and (*S*)-heptyl amino-alcohol quinoline derivatives studied in this work displayed a steady activity against a panel of *P. falciparum* clones with varying resistance profiles to CQ and MQ and from different regional origins. Three major points in favour of a continuing development of these compounds in preclinical studies were (i) their *in vitro* combination with DHA that allowed for addition of the effects or synergy, depending on the ratios tested, and displayed an anti-malarial activity as good as that of the DHA/MQ combination; (ii) *in vivo* survival rates as good as those of MQ, in a mouse-model of *P. berghei* ANKA infection; and, (iii) HC_50_s at least 1,000-fold higher than their antiplasmodial IC_50_s, pointing towards a rather safe use as compared to MQ. Taken together, and even though the precise mechanism of action of these compounds remains to be elucidated, the results obtained here make those two (*S*)-amino-alcohol quinoline derivatives good candidates for the development of new ACT, hopefully with fewer neurologic side effects than those currently marketed ACT including MQ, their structurally close parent.
